# Insights and decision support for home health care services in times of disasters

**DOI:** 10.1007/s10100-021-00770-5

**Published:** 2021-08-02

**Authors:** Klaus-Dieter Rest, Patrick Hirsch

**Affiliations:** Institute of Production and Logistics, University of Natural Resources and Life Sciences, Vienna, Feistmantelstrasse 4, 1180 Vienna, Austria

**Keywords:** Home health care, Disaster management, Causal-Loop-Diagrams, Decision support system, COVID-19 case study

## Abstract

Home health care (HHC) services are of vital importance for the health care system of many countries. Further increases in their demand must be expected and with it grows the need to sustain these services in times of disasters. Existing risk assessment tools and guides support HHC service providers to secure their services. However, they do not provide insights on interdependencies of complex systems like HHC. Causal-Loop-Diagrams (CLDs) are generated to visualize the impacts of epidemics, blackouts, heatwaves, and floods on the HHC system. CLDs help to understand the system design as well as cascading effects. Additionally, they simplify the process of identifying points of action in order to mitigate the impacts of disasters. In a case study, the course of the COVID-19 pandemic and its effects on HHC in Austria in spring 2020 are shown. A decision support system (DSS) to support the daily scheduling of HHC nurses is presented and applied to numerically analyze the impacts of the COVID-19 pandemic, using real-world data from a HHC service provider in Vienna. The DSS is based on a Tabu Search metaheuristic that specifically aims to deal with the peculiarities of urban regions. Various transport modes are considered, including time-dependent public transport.

## Introduction

Many countries are experiencing a significant increase in demand for long-term care. The average share of the population aged 65$+$ years in OECD countries increased from about 9% in 1960 to 17% in 2015, and is forecast to reach 28% in 2050. The share of those aged 80+ years is expected to grow even stronger. An increase in life expectancy does not mean that the extra years are lived in good health. In fact, the risk to develop disabilities and to need assistance also increases. On average across the OECD countries, 13% of people over 65 receive long-term care and more than half of them are 80$+$ years old (OECD 2017). Many care-dependent people are cared for by friends or relatives. Others receive professional help either in nursing homes, day-care centers, or their own homes through home health care (HHC) services. As outlined in Rest et al. (2012), HHC services allow old and frail people to stay as long as possible in their familiar environment but still receive professional care. Furthermore, the HHC system is also more cost efficient than institutional long-term care.

HHC service providers offer a wide range of services, ranging from assisting in daily life to qualified medical services. People with limited mobility or medical needs (e.g., diabetics) require consistent services to avoid a deterioration of their health. But even seemingly less important tasks like assisting in daily life are of vital importance. These activities deal not only with personal hygiene or preparing meals, they are also used to monitor the health of the people. This information is crucial to adapt the services to the changing needs. Expecting an increase in demand and being a vital part of the health care system, HHC services must be sustained by all means in order to avoid health implications. To maintain business continuity, and thus continuity of care, HHC service providers must prepare for a variety of disaster situations.

The aim of this paper is to analyze the effects of natural and technical disasters on HHC services in urban regions. Based on information from the Austrian Red Cross (ARC), one of the major HHC service providers in Austria, epidemics, heat waves, floods, and blackouts have been defined as most important. Initial analysis and results have been published in Rest et al. (2012) and in the conference proceedings of Rest and Hirsch (2015). These findings are extended in two ways. First, the concepts of System Dynamics (SD), especially Causal-Loop-Diagrams (CLDs), are used to visualize and identify influential factors and vulnerabilities of HHC services. Second, in a case study the impacts of the pandemic of the coronavirus disease 2019 (COVID-19) on HHC in Austria in spring 2020 are analyzed. A decision support system (DSS) for the daily scheduling of HHC nurses is presented and applied to analyze the impacts of the COVID-19 pandemic. The DSS is a commercially available advancement of the previously developed algorithms, published in Rest and Hirsch (2016). In contrast to existing work, the DSS focuses on urban regions and allows planning with public transport and with time-dependent travel times. It is applied to real-world data from a Viennese HHC service provider to show the effects of the strict COVID-19 actions. As different transport modes are considered, their impact on the planning is outlined as well. The main findings of this work aim to raise the awareness of HHC service providers regarding their vulnerabilities. The DSS itself can be used for the daily scheduling as well as for training and capacity analysis and planning. During a disaster, it guarantees that the available staff is scheduled as efficient as possible.

The remainder of the paper is organized as follows: Sect. 2 discusses emergency preparedness of HHC services and relevant literature in this field. Section 3 assess the vulnerability of critical assets of HHC to the four mentioned disasters and plots them in CLDs. In Sect. 4, the COVID-19 case study is presented, including the presentation and application of the DSS using real-world data from a Viennese HHC service provider. Final remarks and an outlook on future research are given in Sect. 5.

## Emergency preparedness of HHC services

HHC provides unique capabilities to manage disaster situations, both before and during the event. The ability to deliver health services in non-structured environments makes them ideal as key responders in times of crisis (NAHC 2008). Identifying and addressing the needs of vulnerable people has been the focus of several studies (e.g., Aldrich and Benson 2008; Khorram-Manesh et al. 2017). HHC services are tailored to the needs of care-dependent people, a group mainly consisting of frail older adults and people with disabilities. Exactly this group is disproportionately affected by emergencies. According to Aldrich and Benson (2008), about 80% of older adults have at least one chronic condition that makes them more vulnerable during a disaster. They point out that, for example, 71% of the fatalities of the hurricane Katrina in 2005 were over the age of 65 and that the median age of the heat-related deaths during the heat wave in Chicago 1995 was 75 years. Thus, HHC services already have direct access to one of the most vulnerable groups, which provides in-depth knowledge of their medical needs, impairments, resources, as well as their home environments. In addition, it enables targeted dissemination of public health (e.g., about vaccinations) and disaster preparedness information.

A manual for developing an all-hazards emergency preparedness plan is provided by the NAHC (2008). It lists potential hazardous events that should be rated based on probability, vulnerability and preparedness. Furthermore, a variety of recommended actions are given to increase the resilience of HHC services. In scientific literature, different approaches are proposed to increase the emergency preparedness of HHC services. Wyte-Lake et al. (2015) present the results of a literature review examining HHC organization policies and procedures, lessons learned in the field, and expert recommendations. Most of the literature discusses only individual actions (e.g., patient classification systems) or actions to tackle individual disasters (e.g., avian influenza). All-hazards risk assessments are only addressed by Doherty (2004) and Rodriguez and Long (2006). They provide advice on how to carry out risk assessments, particularly by raising questions that need to be addressed by the HHC service providers. However, the HHC system as well as various disaster events are complex systems that involve numerous interdependencies. In our opinion, these interdependencies have received little attention in the existing literature and risk assessment tools, but can be tackle by applying SD methods.

## Vulnerability assessment and systems thinking

**Fig. 1:**
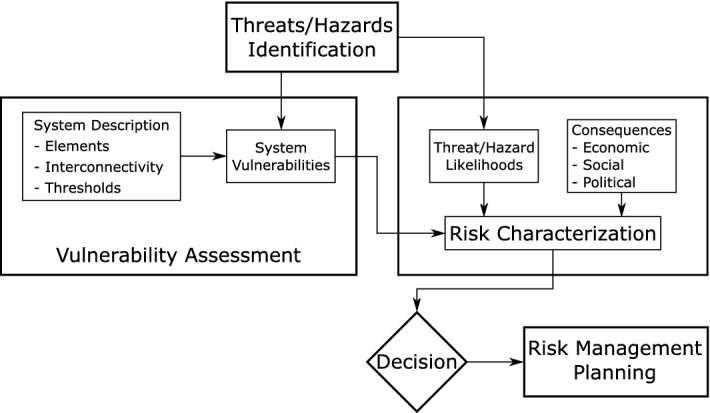
Risk assessment process according to Baker (2005)

Risk assessments are an important part of the risk management procedure and a precondition for the subsequent phases of the crisis management cycle (Pursiainen 2017). As shown in Fig. 1, the first step of a risk assessment and management process is to identify relevant threats and hazards. In the next step, vulnerability assessments are used to evaluate the weaknesses of a system regarding these threats. Afterward, the risks are determined by assigning likelihoods to each threat. The final step consists of managerial decision making, heavily influenced by the resulting risk characterizations and the financial possibilities of an organization. The focus of this paper lies on the vulnerability assessments as we think that the existing HHC literature and risk assessment tools are deficient in this area. While they provide great guidelines for the remaining steps, they offer little help in terms of insights into interdependencies. According to Baker (2005), the main objectives of vulnerability assessments are to...

- understand the organization’s mission and mission-supporting systems.
- identify mission-threatening vulnerabilities of critical systems.
- understand system design and operation to determine failure modes.
- identify consequences of failures and cascading effects on other systems.

Understanding complex systems is the main goal of the SD methodology. To the best of our knowledge SD methods have not been applied yet to model the HHC system. SD has been described first by Forrester (1997) to model the behavior of complex systems that are characterized by interdependencies of the influencing variables. CLDs are one of the main concepts in the SD toolset and used to visualize cause-and-effect relationships and feedback processes. A system is modeled as a network consisting of nodes representing variables and links representing an interaction between two variables. A link of a positive (resp. negative) interaction means a change in the same (resp. opposite) direction, i.e., if the cause increases then the effect increases (resp. decreases). Powell et al. (2016) show the advantages of using CLDs during the risk assessment process and use them to analyze flood threat to an electricity substation. CLDs are also well suited to visualize cascading effects of disasters, as shown by Berariu et al. (2015). They analyze cascade effects of floods and heatwaves and their impact on critical infrastructures.

Expert interviews with decision makers of the ARC identified epidemics, blackouts, heatwaves, and floods as their most significant disaster events. The ARC is not only one of the largest HHC service providers in Austria, but also has extensive expertise in health care and disaster management. The effects of these disasters have been analyzed on the basis of scientific publications, addressing general disaster impacts on critical infrastructure and lessons learned from specific events. The findings were modeled in the form of CLDs and discussed and refined again with the ARC. In the following, the CLDs for each of these disasters are presented.

### Epidemics

Infectious diseases repeatedly affected large parts of the population within a short period of time. Regionally confined outbreaks are classified as epidemics while the term pandemic describes a worldwide spread. Gershon et al. (2007) state that more than 30 novel pathogens have been identified in the past 2 decades and that the incidence of emerging pathogens is increasing. Globalization, with its increase in international travel, fosters the spread of diseases and areas of high population density are particularly at risk. For example, the 2002 SARS outbreak started in China and spread rapidly to Toronto, London, and New York. The US Centers for Disease Control and Prevention estimates that the 2009 H1N1 (swine flu) outbreak resulted in about 60 million cases, 270,000 hospitalizations, and 12,500 deaths in the United States (CDC 2019). At the time of writing, the ongoing COVID-19 pandemic has already caused massive economic damage and social restrictions worldwide. As of March 20, 2021, the COVID-19 Dashboard of the Johns Hopkins University reports more than 122 million infections and more than 2.7 million deaths across 192 countries and territories (Johns Hopkins University 2021). The pandemic is described in detail in the COVID-19 case study in Sect. 4.

**Fig. 2:**
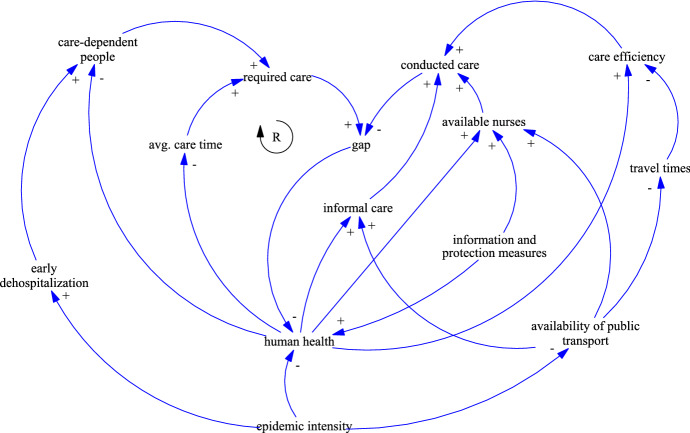
CLD of the disaster impacts of epidemics on HHC

The CLD in Fig. 2 shows the impact of epidemics. The intensity of an epidemic directly affects the variable *human health*, which expresses the health of the population and thus, the health of the nurses, the care-dependent people and their relatives. When affected by the disaster, the overall health of the population decreases. This decreases the number of *available nurses*. At the same time, people in poor health require more care and those whose health condition previously allowed for a self-sufficient life may now need care. The relation of vulnerable groups requiring additional care during times of disasters is emphasized by several studies (e.g., Fernandez et al. 2002; Khorram-Manesh et al. 2017). In addition, Knebel and Phillips (2008) point out that the number of care-dependent people will also increase because more people are discharged earlier from hospitals, due to short capacities. This is depicted in the CLD by *early dehospitalization*. Thus, it can be concluded that the better the *human health*, the lower the number of *care-dependent people* and the lower the *average care time*. The *human health* is also an influential factor of the *care efficiency*. Both, the physical and psychological health highly influences the efficiency of the conducted care.

Another major problem arises from the nurses’ ability and willingness to work. They might be sick themselves or have to care for other family members like children (e.g. due to the closure of schools and day-care centers). Knebel and Phillips (2008) estimate that about a quarter of the nurses will be sick themselves during an influenza epidemic. The willingness of healthcare personnel to work during different disease outbreaks (e.g., avian influenza and pox) has been addressed by several studies (e.g., Mackler et al. 2007; Irvin et al. 2008). They conclude that without protective equipment or vaccinations, few are willing to show up for duty, and even fewer if they fear that they might spread the disease to their own family members. However, the willingness to work increases with the staff’s qualification and level of information about the disease. Thus, the variable *information and protection measures* directly affects not only *human health* by preventing infections, but also the *available nurses*. The availability of transportation also affect the nurses’ ability to work. Fuel supplies and public transport might be limited because of high absenteeism or shut downs in order to limit the spread of the disease (Knebel and Phillips 2008). The higher the *availability of public transport*, the lower the *travel times*. The *travel times* are inversely proportional to the *care efficiency* because the more time is spent for traveling, the less is available for caring. A significant portion of care is still conducted by relatives and denoted by the variable *informal care*. It is directly proportional to *human health*. The worse the *human health*, the lower the amount of *informal care*. Relatives are unable to carry out care activities for the same reasons as the nurses. However, they are more affected as they might not have access to protective equipment or vaccinations to protect themselves from infection.

The main challenge of epidemics can be summarized by a huge increase in the number of care-dependent people and service times, paired with a significant decrease of the availability of nurses and informal care. The efficiency of the conducted care services is also expected to decrease. A higher demand for HHC services (*required care*) is thus offset by a reduced care potential (*conducted care*). The resulting *gap* creates a reinforcing feedback loop as unsatisfied demand decreases the *human health* of the care-dependent people. As a consequence, additional care is needed, which again increases the gap.

### Blackout

**Fig. 3:**
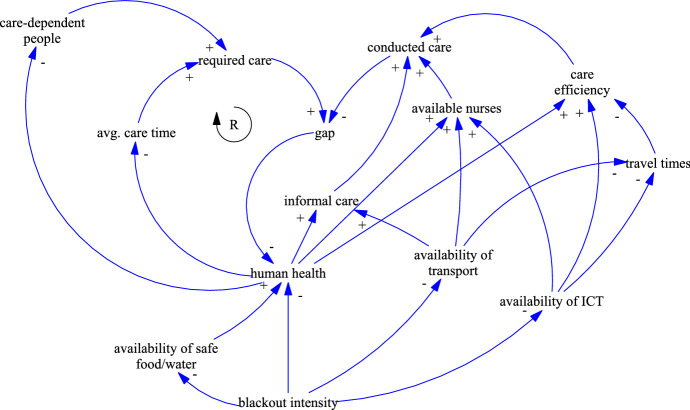
CLD of the disaster impacts of blackouts on HHC

Blackouts refer to large-scale power outages that last for several days or even weeks. However, even short-time interruptions of the electricity supply might result in cascading failures of critical systems. They are often the cascading result of other events (e.g., floods or winter storms) or of man-made- and technical failures. Alhelou et al. (2019) published a survey on blackouts around the globe and their cascading events. Europe in general, and Austria in particular, experience a very high level of electricity supply security. The single European electricity market imposes both, threats and benefits to supply security. Local shortages or disruptions might be compensated transnationally to avoid serious incidents, but in the worst case, they have ripple effects on their surrounding area, leading to large-scale power outages. For example, in September 2003, a tree flashover at a high-voltage line in Switzerland triggered a sequence of events that led to a separation of Italy from the European grid. Italy suffered a nationwide blackout that took about 19 h to re-energize all regions (Alhelou et al. 2019). According to Marston (2018), the US power supply suffers from its aging infrastructure and by the diverse set of infrastructure owners and operators, making the US power system even more susceptible to blackouts. The author also describes the impacts of various environmental and human-related threats, like physical sabotage and cyberattacks on the different electric system components (e.g., generation, transmission, or distribution). The significant growth of electricity generation from renewable energy sources results in an increasing supply volatility, thereby putting pressure on transmission and distribution systems (Reichl et al. 2013). The flourishing demand for electric vehicles will additionally stress the electricity supply. The frequency and scale of blackouts might therefore increase. Figure 3 visualizes the impacts of a blackout on the HHC system.

Blackouts affect nearly every aspect of daily life. Alhelou et al. (2019) describe the social, economic, and political impacts of blackouts on modern societies. For HHC, the most important impacts are on telecommunications, transport, and the health care sector. Most HHC service providers rely heavily on their IT and communication systems. Schedules are usually sent electronically to mobile devices of the nurses. These devices are also used to track and monitor the conducted care services directly at the clients’ locations. Cowie et al. (2004) examine the impact of two widespread blackouts on the internet communication. Only the internet backbones were unaffected and thousands of institutional networks and millions of internet users where offline for hours or days, including banks, companies, hospitals and government institutions. Without IT systems and with limited ways of communication (modeled by *availability of ICT*) the *available nurses* are expected to decrease. Furthermore, the scheduling of the staff is less efficient, resulting in longer *travel times* and a reduced *care efficiency*. A blackout also directly affects the *availability of transport*. Electricity-based transport (e.g., underground, train, and tram) will be inoperable and buses and cars suffer from lack of fuel and the failure of traffic management systems (e.g., traffic lights). A lack of *availability of transport* leads to longer *travel times* and to a reduction of *available nurses* and *informal care*.

The impacts on the health care sector are well documented by previous incidents. For example, the blackout that hit the northeast of the United States and the Canadian province of Ontario in August 2003 lasted for several days. Freese et al. (2006) reveal that the number of emergency calls increased by 103% in New York during this time. As the blackout happened in summer, most medical emergencies were related to cardiac and respiratory complaints. Beatty et al. (2006) emphasize the critical situation of those people, relying on electric equipment (e.g., lifting or oxygen devices) or medicines that require constant refrigeration. The authors also address an increase of foodborne diseases as well as contamination with untreated sewage. Klein et al. (2005) present the lessons learned from hospitals’ perspectives. Compared to HHC, hospitals are well controlled environments and well prepared for disasters. Nevertheless, serious problems were encountered, including lighting, water supply, sanitation, hygiene, heating, ventilation, and air conditioning. Most of these issues were related to failing generators and city-wide loss of tap water supply and sewage disposal. The authors also identified staffing problems due to lack of communication, transportation, and childcare.

It can be concluded, that blackouts decrease *human health* in various ways, but most harm is done by the loss of electrically powered (medical) equipment or by the occurrence of diseases. The *availability of safe food/water* decreases without electricity, which further reduces *human health*. Especially cities with many high-rise buildings are at risk and depending on the season, the lack of heating or air conditioning further aggravates these adverse health effects. The lower *human health*, the higher the number of *care-dependent people* and the higher the *avg. care time*. On the other hand, the *informal care* potential, the number of *available nurses* and the *care efficiency* are reduced. The top part of the CLD shows the same reinforcing feedback loop as during epidemics. Unsatisfied demand for care decreases the health of the care-dependent people, leading to an even higher demand.

### Heatwave

**Fig. 4:**
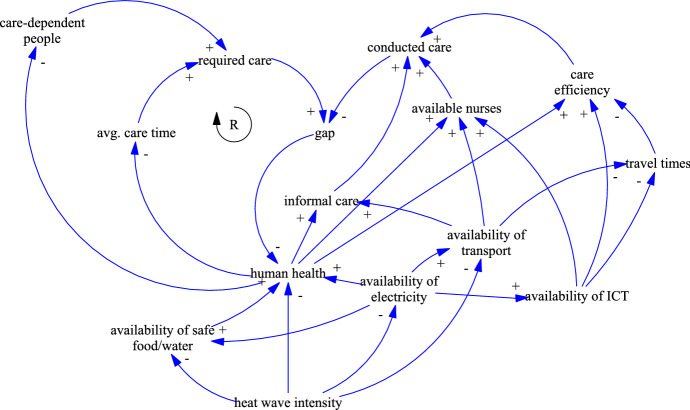
CLD of the disaster impacts of heatwaves on HHC

Heatwaves refer to continuous periods with high temperature that last for weeks. Because of climate change and global warming, the frequency and intensity of heatwaves is expected to increase. Chapman et al. (2013) outline that urban regions are particularly affected by heatwaves, due to the urban heat island effect, which describes that temperatures in city centers are up to 10 $^\circ$C higher than in the surroundings. Cities not only produce and absorb more heat, they also store it longer and therefore, they cool off more slowly. The CLD for heatwaves, shown in Fig. 4, resembles the CLD of blackouts in Fig. 3. The effects of both disasters are not only similar, but blackouts are often the cascading result of heatwaves. The main difference is therefore, that the CLD for heatwaves has been extended by an additional variable modeling the *availability of electricity*. This variable corresponds to the previous *blackout intensity*.

The impacts of heatwaves on *human health* are well documented and several studies outline the causal relation of increased mortality and morbidity during heatwaves. Mayrhuber et al. (2018) recently published a review on this topic in order to assess the vulnerability across societies. They emphasize that elderly and chronically ill people are among the most susceptible groups at risk. Being confined to bed, not leaving home daily, and being unable to care for oneself result in the highest risk of death. The most common health implications are heatstroke, dehydration, cardiovascular, and respiratory diseases (Haines et al. 2006). Heatwaves might result in water shortages and thus, in an increase of infectious diseases. Zander et al. (2018) describe the effects of heat stress and relief measures on the workforce. Without relief measures (e.g., cooling, resting, or hydration) high temperatures result in fatigue, headaches and in reduced cognitive abilities and decision quality. The productivity of staff is significantly reduced and the risk of work place accidents increases, thus impacting the *care efficiency* and the *available nurses*.

In addition to the health effects, heat waves also affect the physical infrastructure. McEvoy et al. (2012) studied the impacts of a heatwave on Melbourne’s critical infrastructure. They discovered severe impacts on the road, rail, and electricity infrastructure. The electricity generation is reduced during heatwaves. Hydro power stations suffer from low water levels and the efficiency of steam turbines depend on the temperature difference of the cooling water. However, the demand for electricity increases significantly because of air conditioning. As a consequence, the heatwave resulted in blackouts. More than a third of the train services were canceled because of the electrical faults, buckling rails, and failing air conditioning of the trains. Road traffic was affected by failing traffic management systems and by road bleeding (McEvoy et al. 2012). Similar findings have been reported by Arkell and Darch (2006) for London’s transport network. It can be concluded, that heatwaves decreases both, the *availability of transport* and the *availability of ICT*.

### Flood

**Fig. 5:**
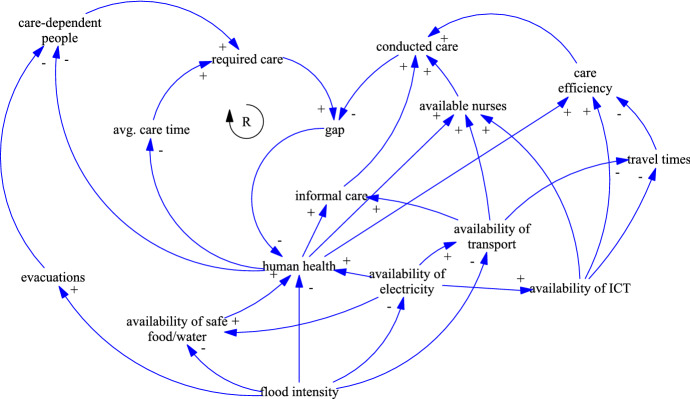
CLD of the disaster impacts of floods on HHC

Different types of floods must be distinguished. River floods are usually slow-rising and more predictable, providing time to prepare preventive actions. The predictability of coastal floods depends on the cause. Heavy storms or hurricanes are well monitored and allow for several days of preparation. Tsunamis however, are caused by spontaneous events like earthquakes and provide only a few hours of preparation, at best. The least predictable are flash floods, usually caused by heavy rain. Alpine and urban regions are especially at risk as they have large areas of sealed surfaces. Figure 5 shows the CLD of flood impacts on HHC. Again, this CLD has many similarities with the CLD of blackouts, because floods often cause widespread blackouts by flooding electrical equipment. However, *evacuations* have been included in the CLD. Usually, residents are not permitted to stay in their homes if they are flooded or even threatened to be flooded in near future. They often stay with relatives or at emergency shelters and thus, might not need HHC services during this time. The more people evacuated, the lower the number of *care-dependent people* in HHC.

The main impacts of floods are on *human health*, *availability of transport*, and *availability of ICT*. The health impacts of floods have been analyzed by Jakubicka et al. (2010). They distinguish short-term effects (e.g., injuries, or an increase in waterborne diseases) and long-term effects (e.g., chronic disease, or mental health issues). The disruption of sewage disposal and water treatment infrastructures has been identified as one reason for disease outbreaks. Mental health impacts are based on the destruction of property, loss of life, geographic displacement, or anxiety about event recurrence. Suk et al. (2019) published a literature review to show how cascade effects of floods results in outbreaks of infectious diseases. According to Haines et al. (2006), large parts of the city of Dresden lost electricity and fresh water for several days during the flood that hit Europe in 2002. For the city of Vienna, floods are a high-impact low-probability risk because of large-scale flood protections (e.g., Danube Island). However, if these protections fail the effects are catastrophic, as outlined by the flood risk analysis of Compton et al. (2009). The transport infrastructure would be heavily damaged because of possible flooding of subway lines. The authors refer to subway floodings in Boston, Seoul, Taipei, and Prague, which took them out of service for several months. Road traffic concentrates itself on the remaining, non-flooded roads, leading to congestion and prolonged *travel times*.

## Decision support in times of disasters: a COVID-19 case study in Austria

In late 2019, a novel coronavirus infection rapidly developed into an ongoing worldwide pandemic. The main transmission takes place from person to person, either through respiratory droplets (e.g., breathing, sneezing, coughing) or (in)direct contact with an infected person. The infection mostly affects the respiratory tract, with symptoms ranging from those of a common cold to very severe respiratory infections. While most infected people show only mild symptoms, older people and those with pre-existing conditions are particularly at risk. As a result, more than half of all COVID-19-related deaths in most EU countries are attributable to elderly residents of long term care facilities and nursing homes (ECDC 2020). The World Health Organization declared the outbreak as a pandemic on March 11, 2020 (Kreidl et al. 2020). As of March 20, 2021, the COVID-19 Dashboard of the Johns Hopkins University reports more than 122 million infections and more than 2.7 million deaths across 192 countries and territories (Johns Hopkins University 2021). In this section, the course of the COVID-19 pandemic in Austria in spring 2020 is described as well as the actions taken by the government and their effects on HHC. Subsequently, a DSS is presented that aims to support HHC services by optimizing the daily scheduling of HHC nurses. Based on the variables identified in Sect. 3, a case study with real-world data analyzes the impacts of the COVID-19 pandemic on the scheduling of the nurses.

### COVID-19 pandemic and measures in Austria

In spring 2020, there were no specific treatments or approved vaccines for COVID-19. The measures and recommended actions focused on the treatment of the symptoms as well as preventing the further spread of the disease. The first diagnosed infections in Europe (January 24) were associated with travels from or to areas in south-east Asia. At that time, the European countries relied on screening and isolating symptomatic people coming from such an area or who had direct contact with a confirmed case. The first major outbreak in Europe was in Lombardy, northern Italy and thus, in the immediate vicinity of Austria (Moshammer et al. 2020). According to Kreidl et al. (2020), the first diagnosed cases in Austria (February 25) were an Italian couple working in Innsbruck but returning from Lombardy. Two days later, the first infected Austrian residents where diagnosed in Vienna. In retrospect, it became apparent that there were already large clusters of infections in skiing areas in the federal state of Tyrol. Between March 7 and 17 a total of 145 infections were diagnosed and attributed to a ski resort. It is assumed that the crowding conditions in aprés ski bars with infected staff members with mild symptoms during the influenza season resulted in an uncontrolled spread of the virus. On March 12 Austria recorded the first COVID-19 fatality (Kreidl et al. 2020).

Desson et al. (2020) compare the policy responses of Austria, Germany and Swiss in the early stage of the pandemic. With steadily increasing numbers of diagnosed infections and hospitalizations, one of the first actions in Austria was the introduction of selective border controls with health checks on March 6, especially on the Italian border. On March 10, people were encouraged to practice social distancing and to work from home, if possible. Public events like the upcoming Vienna City Marathon have been canceled and public facilities (e.g., museums, federal gardens) were closed (Desson et al. 2020). Universities were announced to close and switch to distance learning by March 16, at the latest. Further measures for schools and limits on the number of people attending events (incl. restaurants) have been promised. On March 12, the day of the first COVID-19 fatality, visits to hospitals were banned and actions were taken to increase hospital capacities (e.g., postpone/cancel elective surgeries) and to establish dedicated COVID-19 treatment centers (Pollak et al. 2020). In addition, about 10,000 citizens completing their mandatory civil service were also moved into health-care support roles as social care workers and paramedics (Desson et al. 2020). As a result of the ski resort clusters, some Tyrolean communities were quarantined for 14 days on March 13. In anticipation of a nationwide curfew, panic purchases took place, overloading the supply chains of certain products. In addition, a ban on visits to nursing homes was announced (Pollak et al. 2020). On March 16, the Austrian government declared a national state of emergency and initiated a strict nationwide lockdown (Desson et al. 2020). Borders were closed, air traffic was largely suspended, shops (apart from basic supply), restaurants, and bars were closed the following day (Pollak et al. 2020). Strict contact regulations and curfews demanded that people leave their home only for four reasons: (1) covering their basic needs (e.g., supermarkets, pharmacies), (2) essential work, (3) assisting other people, and (4) taking walks alone or with people from the same household. As a result of the lockdown, schools and kindergartens were closed and public transportation reduced its operation significantly. HHC nurses who are dependent on public transportation suffered from longer travel times. On March 30, it was announced that a mouth nose protection must be worn when shopping. The lockdown was gradually lifted on April 14, due to the successful containment and its economic impacts. Smaller shops were allowed to reopen and the requirement to wear a face mask was extended to public transport. On May 1, the curfews ended and the remaining shops and body-related service providers (e.g. hairdressers) reopened. Two weeks later, restaurants, bars, and cafes opened with restricted numbers of visitors. Schools opened nationwide on May 18 (Pollak et al. 2020).

Schmidt et al. (2020) analyzed the impact of COVID-19 on users and providers of long-term care services in Austria. On May 15, Austria had a total of 16,068 confirmed COVID-19 cases and 628 attributed fatalities. 788 residents of nursing homes and 448 staff were tested positive as of May 6. While 28% of the infected residents died, the number of cases in nursing homes is estimated to be low in comparison with other countries. It is outlined that the Austrian long-term care system significantly relies on migrant carers from Eastern European countries, who were heavily affected by travel restrictions and border closures. To prevent staff shortages, staffing regulations have been loosened to allow people with limited (e.g., in training) or no qualifications to provide basic care. To avoid infections of the nurses and transmissions to clients, recommendations for preventive and protective measures were published. However, it was difficult to provide sufficient amounts protective gear during the first weeks of the pandemic. In order to sustain the informal care potential, telephone hotlines providing psychological counseling and self-help through online support networks (e.g., online courses for unpaid carers) were provided (Schmidt et al. 2020). With reference to the CLD for epidemics presented in Sect. 3.1, the mitigation measures and their consequences are well locatable in the CLD. They mainly relate to *human health*, *available nurses*, *care-dependent people*, and *availability of public transport*.

### Decision support system

A DSS can support multiple phases of the disaster management cycle (mitigation, preparedness, response, and recovery). For example, it allows HHC service providers to determine their operational limits and to adjust their processes and capacity planning, in order to enhance the resilience of their organization. Furthermore, a DSS can be used for the training of the dispatchers and nurses to prepare for various disaster events. During the response phase of a disaster, it guarantees that the available resources are used as efficiently as possible. By speeding up the planning process, a DSS also frees time of the dispatchers for other important activities.

Applying operations research methods and techniques to support HHC received a lot of attention in the recent years. The organization and processes of HHC service providers differ even within a single country, resulting in a wide range of publications, addressing different aspects of HHC. The literature review of Hulshof et al. (2012) lists various decision problems in HHC on the strategic (e.g., districting, capacity dimensioning), tactical (e.g., capacity allocation, staff-shift scheduling), and operational level (e.g., staff-to-visit assignment, route creation). Optimizing the routing of HHC services, which is in the main focus of this section, is addressed in the comprehensive literature reviews of Fikar and Hirsch (2017) and Grieco et al. (2020). They reveal various challenging routing problems with a wide range of regulative and operational constraints as well as diverse objectives. Despite the large number of works, the impacts of disaster scenarios on HHC services have been hardly discussed. To the best of our knowledge, there is only a limited number of publications to support HHC in such times. Barkaoui et al. (2018) developed a mixed integer linear program with a dynamic risk-based clustering. The model considers the geographical proximity of the clients and each client’s predefined risk rating for each period. It is decided which groups are evacuated and which HHC resources are assigned to the groups of clients staying at home during forecastable natural disasters such as floods. The routing of the nurses is not considered. Trautsamwieser et al. (2011) present a mixed integer linear program as well as a Variable Neighborhood Search-based metaheuristic to support the daily routing of HHC nurses in the rural area of Upper Austria. A sensitivity analysis is carried out to show the impacts of natural disasters on the planning. A real-world flood is analyzed as well as official flood risk scenarios with a 30, 100, and 200 year return period.

The solution approach of the presented DSS and the underlying HHC routing problem are based on those published in Rest and Hirsch (2016). They have been further developed by the same authors into a commercially used software that has been marketed by the *ingentus decision support KG*, a spin-off of the Institute of Production and Logistics of the University of Natural Resources and Life Sciences Vienna. The DSS is used by a major HHC service provider in Vienna for their daily planning. In total this service provider has about 750 nurses and over 3000 clients. The HHC routing problem has a daily planning horizon and can be briefly described by the characteristics of the clients and nurses.

Clients require one or more services (jobs) per day and each job...

- must be executed by a feasible and appropriately skilled nurse (i.e., qualification level, language skills, sex, not excluded nurse or transport mode).
- has to start within its given (hard) time window.
- has a fixed duration that must not be shortened.
- should be assigned to the clients’ team of preferred nurses and all of his/her jobs should be assigned to the same nurse (for each qualification level).
- should be planned so that the minimum and maximum time offsets between jobs are fulfilled (e.g., 2h between a morning and lunch job).
- that is marked as ’multiclient job’ has to be carried out together with its counterpart (i.e., same time and nurse).

Nurses are required to...

- carry out jobs that correspond to their primary qualification or that require a qualification level that is included in their set of allowed qualifications (results in over-qualification).
- obey working time restrictions (i.e., earliest/latest and minimum/maximum working time).
- hold breaks if the working time exceeds a certain time.
- work at most two shifts a day.
- use one of the available transport modes (i.e., public transport, cars, bicycles, walking).
- start/end their shift either at a depot or directly at the location of their first/last job and return to a predefined location if working two shifts.
- not exceed the total overtime limit defined for all nurses.
- have a similar workload compared to the other nurses (relative to their target working time).

A weighted objective function balances the opposing goals of minimizing the route lengths and maximizing the satisfaction of the clients and nurses. The client’s satisfaction is mainly determined by the consistency of care regarding the visiting times and nurses. For the nurses, overtime and over-qualification are crucial indicators for their satisfaction. A total of 11 soft constraints are used to configure the tradeoffs between the individual factors of the objective function. The actual weights have been set together with the dispatchers and the managerial decision makers of the the HHC service provider. In contrast to the algorithms published in Rest and Hirsch (2016), the DSS must always present a schedule to the dispatcher. For this reason, only a few hard constraints are considered. These consist of assignment constraints that can be evaluated before the actual optimization. If the preliminary checks detect unassignable jobs, the reasons for the infeasibilities are reported. In addition, the time windows of jobs, the start and end times of a nurse’s shift as well as his/her total working time limit can be marked as a hard constraint by the dispatcher. However, if no satisfying solution is found, the best infeasible solution is reported.

The presented HHC routing problem mainly distinguishes itself from other work by the fact that public transport is considered. Public transport operates on timetables that have different departure intervals and travel times during the day. Thus, their time-dependent travel times are modeled using a travel time matrix for each minute of the day. The DSS is able to process timetable data from public transport service providers in the General Transit Feed Specification (GTFS) format, developed by Google. Another unique feature are the mandatory breaks, which in contrast to previous work, can be split into smaller parts. To the best of our knowledge, there are still no other publications except for Rest and Hirsch (2016) in the field of HHC routing, addressing one of these features. The routing itself is based on OpenStreetMap (OSM) data, which is a viable alternative data source, especially in urban regions. The benefits of community gathered data also ensures a better availability of routable maps in the event of a disaster.

Time and efficiency are crucial for practical applications. Due to the complexity of the routing problem, the DSS uses a Tabu Search metaheuristic. The algorithm is explained in detail in Rest and Hirsch (2016). Its process can be summarized as follows: First, an initial solution is generated with an insertion heuristic, based on the centered time windows of the jobs. Afterward, neighbor solutions are generated by moving individual jobs from one nurse to the routes of all other nurses. The insertion into the new routes are based on the ’best insertion’ principle. During the evaluation of the new routes, their starting times are optimized and breaks are inserted, if needed. The route with the best objective value updates the current solution and the cycle of generating new neighbor solutions continues until a termination criteria (i.e., elapsed time, number of iterations without improvements) is met. The best found solution is tracked during the search and returned at the end. The search process temporarily allows infeasible solutions and uses a ’tabu list’ to prevent the reversal of moves for a certain amount of iterations. To further guide the search, dynamically adapted penalties are added to the weighted objective function.

The presented algorithm is based on the same concepts used in Rest and Hirsch (2016). However, several problem specific elements have been changed. The main difference is the optimization of the start time of the nurses. In Rest and Hirsch (2016) the aim was to determine the start time that results in the shortest working time of the nurse. However, this might lead to considerably postponed start times, which leave hardly any leeway for complications. Therefore, the algorithm of the DSS first calculates the earliest possible end of the tour and then determines the latest start for this end. Additional constraints have been implemented to cover the new requirements regarding the multiclient jobs and the minimum and maximum time offsets between jobs. A non-linear constraint was added to seek a balanced workload of the nurses.

**Fig. 6:**
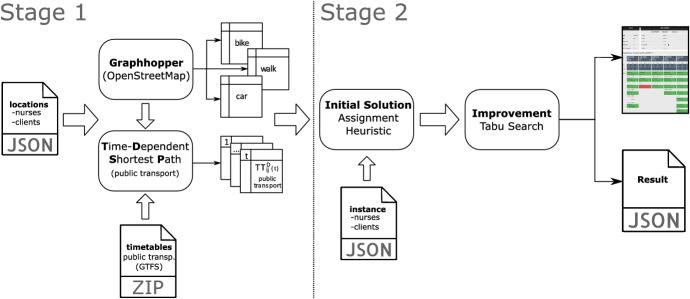
Structure of the DSS

For easy integration in existing systems, the DSS has been developed as a web service using Java 8. This way, it is highly scalable to manage heavy workloads as well as highly customizable and expandable with regard to constraints and objective functions. It is able to solve real-world sized instances with about 250 jobs and 40 nurses within a few minutes. The DSS can be used to evaluate or improve the manual planning of the dispatchers or to compute schedules from scratch. The structure of the DSS is shown in Fig. 6. For data protection reasons, a 2-stage process is followed. In the first stage, geographical data is processed into time-dependent (i.e., public transport) and time-independent (i.e., car, bike, walking) travel time matrices. Following the principle of data minimization, only the final travel time matrices are stored, preventing direct geographical traceability. In the second stage, the travel time matrices and the submitted anonymized instance data are used to compute the schedule. The results are returned as a report (Html format) and as raw data (Json format) in order to display and further process the schedules in the existing software systems of the HHC service provider.

### Numerical studies

Based on the findings in Sects. 3.1 and 4.1, several strategies were followed to numerically assess the impact of the COVID-19 pandemic on HHC in Vienna. The focus was laid on two areas, the transport infrastructure (travel times, availability of transport modes) and the clients (duration of care). The DSS was adapted so that changes in these areas can be done directly through input data modifications. Due to the sensitivity of the real-world data, artificial manipulations such as adding and removing clients, jobs or nurses were avoided. Unfavorable decisions can quickly lead to useless instances whose results may even lead to wrong conclusions. For example, it is easy to overburden some nurses while others hardly work at all because of exclusions. In practice, dispatchers would then deploy nurses in or from different areas, but such decisions are based on the expertise of the dispatchers. The specific settings of each scenario are described below, together with their results.

The DSS is applied to real-world data from a major HHC service provider in Vienna. A total of 16 instances ($I_1-I_{16}$) from regular weekdays are available. For data protection reasons, the instances originate from a corresponding period in 2019. Geographically, the clients are spread across all districts of Vienna. Each instance represents a group of nurses working in a certain area. The zoning has been done by the HHC service provider, primarily based on public transport hubs. The working times of the nurses are given by their contracts and the rosters of the corresponding weekday. Table 1 shows for each instance the number of jobs, nurses, total shifts as well as how many nurses use public transport or cars. The average walking time between all clients is given as indicator for their geographical distribution. The walking speed is based on 5 km/h, for cycling a speed of 18 km/h was assumed and for cars, the speed is based on the OSM road types but set slightly below their limits. However, all speeds are reduced by the actual speed limits on the streets. The qualification level of all nurses and jobs correspond to those of home helpers. The service times of the jobs vary between 15 and 165 min, averaging 49 min. The average length of the jobs’ time windows is just under 2.5 h, but with a minimum length of 15 min and a maximum of 6.75 h for less time critical jobs.

**Table 1:** Data characteristics of the real-world instances

	Jobs	Nurses	Shifts	Pub. transp.	Cars	Avg. walking
	(#)	(#)	(#)	(#)	(#)	(min)
$I_1$	139	28	34	21	7	32
$I_2$	140	20	26	20	0	20
$I_3$	138	18	27	18	0	19
$I_4$	123	20	24	15	5	26
$I_5$	134	18	24	15	3	33
$I_6$	109	18	22	7	11	60
$I_7$	133	24	33	20	4	34
$I_8$	136	27	35	23	4	22
$I_9$	135	21	29	18	3	22
$I_{10}$	129	21	25	14	7	26
$I_{11}$	154	26	33	20	6	47
$I_{12}$	163	21	29	17	4	48
$I_{13}$	121	17	21	16	1	33
$I_{14}$	140	25	32	18	7	32
$I_{15}$	122	26	31	26	0	22
$I_{16}$	134	20	25	17	3	34

The weight setting used for the computations of this paper are the same that the dispatchers at the HHC service provider use in practice. In times of disasters, priorities are usually shifting to ensuring the delivery of care. However, the DSS was designed to always report a solution and its constraints have been configured so that the focus is shifting in case of larger violations. For example, at low workloads, the focus is on meeting the preferences of the clients (i.e., preferred nurses, consistency of care). On the other hand, at high workloads, meeting the mandatory working time restrictions is more important. Furthermore, using the real-world setting allows for a good comparability of the results from before and during the pandemic. All computations have been carried out locally on a Lenovo ThinkPad T490 with an Intel Core i7-8565U processor, 16 GB of RAM and running Windows 10 Pro. The computation time limit has been set to 600 seconds per instance.

The first scenario analyzes transport infrastructure impacts. As outlined in Sect. 4.1, only public transport was affected during the pandemic in Austria. In this scenario, each instance is solved using the timetable data from before and during the lockdown. As reference for a timetable without restrictions the day of January 13, was chosen, a normal weekday without COVID-19 measures. For the second date, March 23 was chosen, the same weekday but 1 week after the curfew came into effect. Just looking at the raw GTFS data, it can be seen that there are substantially less connections on this day. The modal split of the nurses, shown in Table 1, remains unchanged in this scenario. Table 2 compares the resulting schedules for all instances before and during the lockdown. As key performance indicators for the travel impacts the total travel times (incl. waiting) as well as the total overtime of each instance are reported. Additionally, these values were summarized to an objective value to show a percentage increase. Overtime is defined as the time that a nurse works outside his/her defined working time window (e.g., 7 a.m. to 2 p.m.). In addition, dispatchers define a target total working time for each shift of the nurse (e.g., 4 h) and exceeding these targets also counts to overtime. This is done to balance the accumulated over- and undertime of a nurse in order to reach his/her contracted working time. As a consequence, the pre corona results already show a considerable overtime for those instances that were submitted with minimum target working times (e.g., $I_{8}$, $I_{9}$, $I_{12}$).

**Table 2:** Real-world scheduling results for each instance before and during COVID-19

	Pre corona			Corona			
	Travel $+$ wait	Overtime	Objective	Travel $+$ wait	Overtime	Objective	Increase
	(min)	(min)	(min)	(min)	(min)	(min)	(%)
$I_1$	1730	231	1961	1957	294	2251	14.8
$I_2$	1937	164	2101	1989	299	2288	8.9
$I_3$	1838	1320	3158	1887	1321	3208	1.6
$I_4$	1231	701	1932	1325	808	2133	10.4
$I_5$	1400	222	1622	1438	282	1720	6.0
$I_6$	1276	323	1599	1299	328	1627	1.8
$I_7$	2033	564	2597	2423	643	3066	18.1
$I_8$	1344	2751	4095	1383	2772	4155	1.5
$I_9$	1292	3632	4924	1340	3684	5024	2.0
$I_{10}$	1340	1291	2631	1461	1333	2794	6.2
$I_{11}$	1851	880	2731	1882	932	2814	3.0
$I_{12}$	1747	2883	4630	1817	2960	4777	3.2
$I_{13}$	1159	1326	2485	1227	1392	2619	5.4
$I_{14}$	1854	429	2283	1886	442	2328	2.0
$I_{15}$	1739	230	1969	1839	369	2208	12.1
$I_{16}$	1360	364	1724	1429	438	1867	8.3
Mean	1571	1082	2653	1661	1144	2805	6.6

It can be seen that the restrictions on public transport result only in small increases in travel (incl. waiting) times and overtime, amounting to an average increase of the objective value of 6.6%. However, individual instances like $I_{1}$, $I_{4}$, $I_{7}$, and $I_{15}$ show increases from 10% up to 18%, which might be deemed already infeasible by dispatchers. The biggest impact is caused by the share of cars in the modal split, in combination with the average distances between clients. As car travels were not affected by the COVID-19 actions, instances with a high share of car users (e.g., $I_{6}$, $I_{14}$) are less affected as long distances are then covered by nurses using cars. On the other hand, nurses relying on public transport suffer even more from its reduced availability the longer the walking distances between the clients are.

The second scenario aims to analyze the operational limits of the different instances during the COVID-19 pandemic. In the CLD for epidemics in Sect. 3.1 it is outlined that clients require more care in such disaster situations. Thus, it should be analyzed how much the service times can be prolonged before capacity problems occur. Therefore, the service times of all jobs are gradually increased in steps of 10%. Determining the feasibility of an instance is difficult to generalize. From the perspective of a dispatcher, even a minor delay at a single job might render the schedule infeasible, if he/she considers it time critical. On the other hand, a slight violation of the maximum working time can still be acceptable in order to guarantee care for all clients. Therefore, the average overtime per nurse and the average time window violation per job are reported as key performance indicators of the schedules. It has already been shown in the previous scenario that the modal split has a large impact on the scheduling. Thus, all computations are carried out also with additional transport modes. The current mix of public transport and cars is used as reference and in case no changes to the modal split are possible. Under the assumption that already existing cars are still available, calculations were made in which all nurses, who previously used public transport, use cars, bicycles or walking. The use of walking can be seen as a worst case scenario, if public transport is shut down completely or if the dispatcher wants to minimize the risk of infections. The calculations with public transport are again based on the timetable data at the time of the lockdown.

**Table 3:** Impacts of transport modes and service times on overtime and tardiness (in min)

	Prolonged service time					
	+ 0%	+ 10%	+ 20%	+ 30%	+ 40%	+ 50%
*Current mix*						
Avg. overtime	53.4	77.4	101.0	131.1	157.2	189.1
Avg. tardiness	5.3	7.7	11.6	17.3	23.2	31.3
*Car*						
Avg. overtime	32.8	54.1	77.6	107.7	134.4	165.4
Avg. tardiness	2.3	5.3	8.6	13.6	19.0	26.9
*Bike*						
Avg. overtime	39.7	62.3	85.7	115.8	143.2	175.0
Avg. tardiness	3.1	6.6	9.7	14.3	20.4	28.4
*Foot*						
Avg. overtime	55.3	79.3	103.4	132.7	159.5	191.3
Avg. tardiness	5.5	7.9	12.1	17.7	23.5	31.6

Table 3 shows the impacts of the considered transport modes and the prolonged service times on the average overtime and tardiness. Regarding the prolonged service times, both the average overtime per nurse as well as the average tardiness indicate an even increase for each transport mode. At first glance, the numbers seem to be manageable by the HHC provider. At the current modal split the average tardiness increases from about 5 min without prolonged service times up to 31 min at +50%. Being late by 5 min is negligible and by half an hour is also most likely acceptable in times of a pandemic, as long as the jobs are not time critical. On the other hand, the average overtime of each nurse increases from 53 min to slightly more than 3 h. The ability to work additional 3 h every day depends on the nurses’ contracted working times. It is most likely not recommended for longer-lasting events like pandemics. Considering the average instance size of 22 nurses and 134 jobs, even without prolonged service times, the COVID-19 situation results in a total overtime of about 19.5 h and a total tardiness of almost 12 h, on average across all instances. Thus, each instance would need 2.5 additional full-term nurses, working 8 h a day, to compensate the additional workload. The advantage of using cars was already apparent in the previous scenario. In comparison with the current modal split, the overtime can be reduced by 23% if all nurses have access to cars. The use of bicycles is inferior to cars due to the lower average speed. Although the DSS is able to explicitly consider times for parking, this feature is not used by the HHC service provider, because the considered speed limits already result in realistic driving times. However, the bicycle results are still significantly better then the current modal split. Furthermore, they are an economically and ecologically viable alternative, and also usable by nurses who do not have a driver’s license. Avoiding public transport only leads to a slight increase of the average overtime by 2% across all scenarios.

## Discussion and outlook

HHC services are rising in importance in the health care systems of many countries and with it grows the need to sustain these services in times of disasters. Risk assessment tools and guides support HHC service providers to secure their services. However, they do not provide insights on interdependencies of complex systems like HHC. CLDs have been used to visualize the impacts of epidemics, blackouts, heatwaves, and floods on the HHC system. They help to understand the system design as well as cascading effects. Additionally, SD simplifies the process of identifying points of action in order to mitigate the impacts of disasters. For example, during an epidemic, protective equipment is crucial as it prevents not only infection of HHC staff and transmission of the disease to clients, but increases their willingness to work. The CLDs also show the importance of informal care, provided by friends and relatives. This outlines the need for a close coordination with them. In case of their unavailability, HHC services need to step in immediately to prevent health issues. On the other hand, relatives might be able to reduce the pressure of HHC services if they are able to take over some tasks.

In a case study, real-world data from a HHC service provider in Vienna was used to show the impacts of the COVID-19 pandemic on HHC in spring 2020. Furthermore, it shows the applicability of the presented DSS in times of disasters, which can be used for the daily scheduling of the nurses to ensure that the limited resources are used as efficient as possible. By speeding up the planning process, it frees time of the dispatchers for other important activities. It also allows HHC service providers to better prepare for disasters and helps to determine the operational limits of the nursing teams, operating in different areas with different characteristics (i.e., distance between clients, availability of public transport). The DSS also shows the effects of using various transport modes. In urban regions, careful planning allows to cover many distances by foot or (electric) bicycles.

While the DSS was used to analyze the impacts of the COVID-19 pandemic in Vienna, it can also be applied to analyze other disaster scenarios, such as those presented in Sect. 3 of the paper. The applicability of the DSS in other regions and countries depends on the organizational requirements of the respective HHC system. However, as the DSS was developed as a commercial software, much attention was paid to flexibility and customizability. Both, the objective function and the constraints can be easily extended and adjusted to the new requirements. Most of the analysis can be done by varying the input data, making the availability of reliable data one of the biggest challenges.

However, the presented DSS has limitations. While supporting HHC service providers, it further increases their dependency on IT systems, which are especially vulnerable during disasters with limited availability of electricity. While it is designed to run on low-powered hardware like notebooks, continuous local backups of the relevant data are required. Furthermore, in its current stage of development, it is assumed that all jobs have to be carried out. At some point during major disasters the operational limits are reached. For these cases, a computer assisted triage system is needed to prioritize the most critical jobs and to carry out as many jobs as possible. Future work should also cover the dynamics of the HHC routing problem. The short computation times of the DSS allows for rapid re-scheduling, but the generated schedules might be entirely different each time. Especially with limited means of communications, it is usually preferable to adapt to the new situation with as few changes as possible.
